# Extended follow-up of ultrastructural remodeling and functional recovery after lamellar macular hole surgery using autologous platelet-rich plasma

**DOI:** 10.1038/s41598-026-58252-0

**Published:** 2026-06-24

**Authors:** Elisa Vanessa Osterode, Lina Sophie Farhoumand, Lara Buhl, Benedikt Schworm, Thomas Christian Kreutzer, Julian Elias Klaas, Felix Hagenau, Siegfried Georg Priglinger

**Affiliations:** 1 Department of Ophthalmology, LMU University Hospital, Ludwig-Maximilians-University Munich, Mathildenstrasse 8, 80336 Munich, Germany; 2 Department of Ophthalmology, University Medical Center Hamburg-Eppendorf, 20251 Hamburg, Germany

**Keywords:** Lamellar macular hole, LMH, Platelet rich plasma, PRP, Vitrectomy, Peeling, Retinal diseases, Eye diseases, Vision disorders

## Abstract

This prospective interventional case study aimed to describe the anatomical and functional outcomes of pars plana vitrectomy (PPV) with adjunct highly concentrated autologous platelet-rich plasma (PRP) in patients with symptomatic, progressive lamellar macular hole (LMH) over an extended follow-up period. 20 eyes of 20 patients were included with a minimum follow-up of 24 months (mean 40.2 ± 11.8 months) and a morphological diagnosis of LMH. 23-/25-gauge PPV was conducted with peeling of the internal limiting membrane (ILM) and epiretinal proliferation (ERP; if present) with intraoperative application of autologous PRP under air or gas tamponade. Combined phacovitrectomy was performed in phakic eyes. Foveal anatomy was restored in all cases at first follow-up. Best-corrected visual acuity (BCVA) significantly improved from 0.36 ± 0.16 logMAR to 0.19 ± 0.24 logMAR at final follow-up (mean 0.17 logMAR, *p* = 0.005). Central retinal thickness (CRT) increased significantly (*p* < 0.001), indicating retinal restoration. Ellipsoid Zone (EZ) defects, initially present in 80% of patients, fully resolved in 62.5% with a significant reduction of defect width (*p* = 0.005). Microperimetry was stable throughout the entire follow-up. Three patients developed recurrent defects possibly attributable to insufficient peeling or postoperative position. No other significant intra- or postoperative complications were recorded. Autologous PRP in LMH surgery is safe and might be an effective adjuvant, potentially offering long-term anatomical stability and functional recovery while reducing the risk of postoperative complications. Those observed effects are exploratory as the cohort size is small and a control group is missing. A main strength of our study is the extended follow-up period which is essential to capture all possible regeneration mechanism.

**Supplementary Information:**

The online version contains supplementary material available at 10.1038/s41598-026-58252-0.

## Introduction

Lamellar macular holes (LMH) occur in the context of pathological posterior vitreous detachment. First described by J. Donald M. Gass in 1975 as an oval, reddish lesion without full-thickness defect in slit-lamp examination, LMH has been reclassified based on morphological parameters with the aid of spectral-domain optical coherence tomography (SD-OCT) by Duker et al. and Hubschman et al.^1–3^. According to the latter—an international panel of vitreoretinal experts—the diagnosis of LMH is based on the following mandatory SD-OCT-criteria: 1) irregular foveal contour, 2) foveal cavity with undermined edges and, 3) signs of tissue loss. Additional OCT findings may be present, such as ellipsoid zone (EZ) defects, epiretinal proliferation (ERP) and a foveal bump. In this consensus-based classification also other non-full thickness macular defects were differentiated such as pseudoholes (PH) and epiretinal membrane-associated foveoschisis (ERM-FS) which is of critical clinical and ultrastructural relevance to evaluate disease progression, spontaneous resolution, and the need for surgical intervention.

From a clinical perspective most LMH cases remain stable without significant progression or functional deterioration, some patients experience a morphological worsening, particularly involving defects in the external limiting membrane (ELM) and the ellipsoid zone, leading to a decline in central visual acuity^4,5^. However, to date no evidence-based treatment recommendation has been defined resulting in controversial therapeutical approaches: some recommend surgical interventions only with restraint and prefer conservative management while others support early surgical intervention to avoid further progression and improve visual outcomes^6–8^. To prevent such functional impairments, ongoing discussions focus on the optimal timing and choice of surgical intervention.

Pars plana vitrectomy (PPV) with internal limiting membrane (ILM) peeling has emerged as first standard surgical approach for LMH. However, concerns have been raised regarding the potential for iatrogenic trauma, particularly in advanced cases where structural integrity is already compromised^9,10^. Modifications to conventional ILM peeling, including fovea-sparing ILM-peeling, LHEP embedding and the adjunctive use of platelet-rich plasma (PRP) have been explored to improve surgical outcomes while minimizing retinal trauma and postoperative complications^11–17^. PRP is rich in cytokines and growth factors such as platelet-derived growth factor (PDGF), vascular endothelial growth factor (VEGF), epidermal growth factor (EGF) and insulin-like growth factor 1 and 2 (IGF-1, IGF-2), etc.^18,19^. After ILM-peeling PRP is applied and thus encounters injured tissue which leads to a secretion of the growth factors and cytokines. This has been proposed to support tissue regeneration by stimulating Mueller cell activation, their migration and tissue remodelling^20,21^. Recent studies suggest that PRP may enhance the success rates of LMH surgery especially in advanced cases but also reduce the risk of postoperative complications while offering a good safety profile^14–17,22^.

To the best of our knowledge only few clinical data with long-term follow-up is available so far for application of autologous platelet-rich plasma in LMH surgery^14–17^.

Therefore, the aim of our study was to provide a long-term overview (herafter termed ‘extended follow-up’) of functional recovery and ultrastructural regeneration after LMH surgery with adjunct PRP over a period of at least 24 months (up to 60 months).

## Results

A total of 20 eyes from 20 patients with symptomatic, progressive lamellar macular hole were included in the study.

Of those 20 patients were 7 (35%) female and 13 (65%) male. At the time of vitrectomy, the patients had a mean age of 72.4 ± 9.3 years (median 75 years, Range 55–85 years). The lens status was evenly distributed, 11 (55%) patients were phakic and 9 (45%) patients pseudophakic. All phakic patients underwent combined phacovitrectomy with phacoemulsification and implantation of an intraocular lens. The mean follow-up time was 40.2 ± 11.8 months (Median 42 months, Range 24–60 months).

An overview of the patient characteristics is given in Table 1. There were no intraoperative complications (e.g. iatrogenic retinal break) recorded.

**Table 1:** Summary of baseline demographic and clinical data M, male; F, female; OD, right eye; OS, left eye; ERP, epiretinal proliferation; EZ, ellipsoid zone; BCVA, best corrected visual acuity; CRT, central retinal thickness; FU, follow-up;

ID	Age [years]	Sex	Laterality	Lens status		Baseline Morphology [yes = 1, no = 0]		BCVA [logMAR]		CRT [µm]		Follow up [months]
				Baseline	Post-op	ERP	EZ Defects	Baseline	Longest FU	Baseline	Longest FU	
1	78	M	OD	IOL	IOL	1	1	0.4	0.2	121	211	48
2	81	M	OS	phakic	IOL	1	1	0.2	0	125	183	24
3	57	F	OD	phakic	IOL	1	1	0.2	0.3	128	224	60
4	67	F	OD	phakic	IOL	1	0	0.2	-0.1	212	242	60
5	65	M	OS	IOL	IOL	1	1	0.4	0.3	152	213	24
6	61	F	OD	phakic	IOL	0	0	0.2	0	230	257	48
7	80	M	OD	IOL	IOL	1	1	0.2	0.3	128	243	48
8	76	F	OS	IOL	IOL	1	1	0.3	0.3	193	150	48
9	78	F	OD	phakic	IOL	1	1	0.4	0.5	179	247	36
10	79	M	OD	IOL	IOL	1	1	0.4	0	201	259	48
11	56	M	OS	phakic	IOL	1	0	0.2	0	153	296	48
12	81	F	OS	phakic	IOL	1	0	0.3	0	199	225	48
13	75	M	OD	phakic	IOL	1	1	0.4	0.3	159	204	48
14	69	M	OS	phakic	IOL	1	1	0.2	0	137	283	36
15	56	F	OS	IOL	IOL	1	1	0.3	0.1	112	271	36
16	73	M	OD	IOL	IOL	1	1	0.4	0.2	213	206	36
17	71	M	OD	phakic	IOL	1	1	0.7	0.1	230	268	36
18	83	M	OS	IOL	IOL	1	1	0.5	0.2	221	220	24
19	78	M	OD	phakic	IOL	1	1	0.7	0.1	122	198	24
20	84	M	OD	IOL	IOL	1	1	0.6	1	114	256	24

### Morphological outcome

**Fig. 1:**
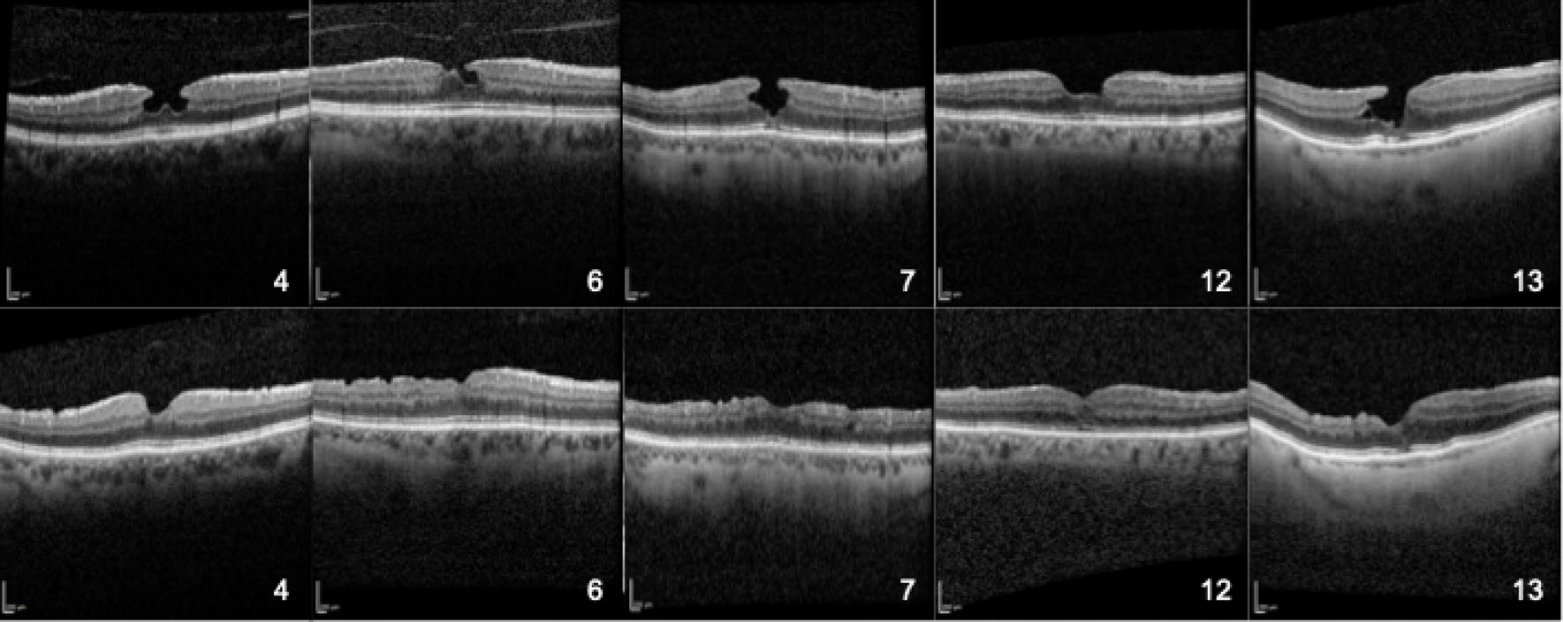
Spectral Domain Optical Coherence Tomography of lamellar macular hole in five patients at baseline (top) and longest follow-up (bottom) at 60 months (ID 4) and 48 months (ID 6, 7, 12, 13).

**Fig. 2:**
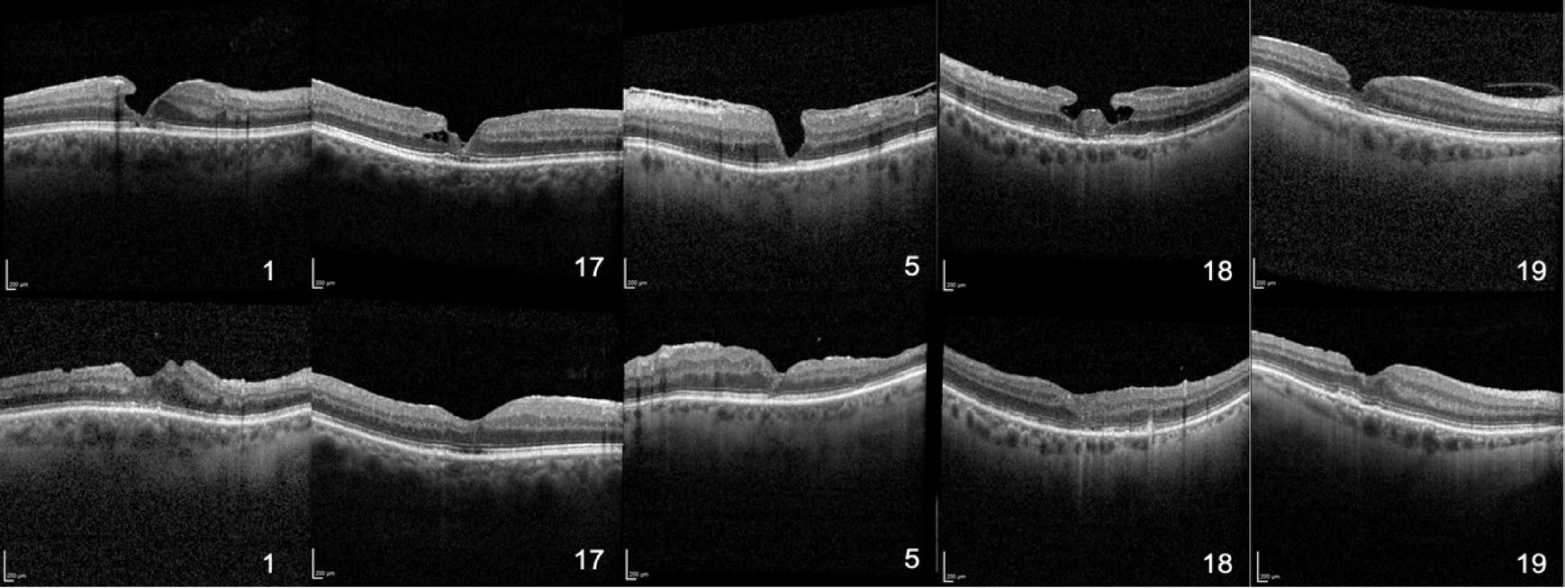
Spectral Domain Optical Coherence Tomography of lamellar macular hole in five patients at baseline (top) and longest follow-up (bottom) at 48 months (ID 1), 36 months (ID 17) and 24 months (ID 5, 18, 19).

All patients initially exhibited primary defect closure without any signs of tissue loss at first follow-up. The foveal morphology remained stable throughout the entire follow-up period (Figs. 1 and 2).

However, recurrences were observed in three cases, which will be described in the following section. One patient had not undergone ILM peeling and showed a recurrent foveal defect 12 months postoperatively. Nevertheless, the patient achieved a postoperative gain in visual acuity of 2 lines, which remained stable as well as the morphology throughout the entire follow-up period (48 months). The second patient showed 24 months postoperatively a deep defect of the inner retinal layers with recurrent epiretinal proliferation. This patient showed already initially a massive amount of epiretinal proliferation which might have not been peeled sufficiently. Despite the defect’s persistence, visual acuity was stable and no deterioration in visual function was noted over the entire follow-up period (48 months). Given this functional stability, a secondary vitrectomy has not been performed to date in those cases. Like already described in our previous data^15–17^. The third patient did not adhere to postoperative recommendations to maintain a supine position, likely resulting in PRP displacement. Consequently, after the endotamponade had resorbed, a second vitrectomy was performed with the reapplication of PRP. Three months later, the foveal morphology had normalized, and functional outcomes showed improvement which was stable since then.

**Fig. 3:**
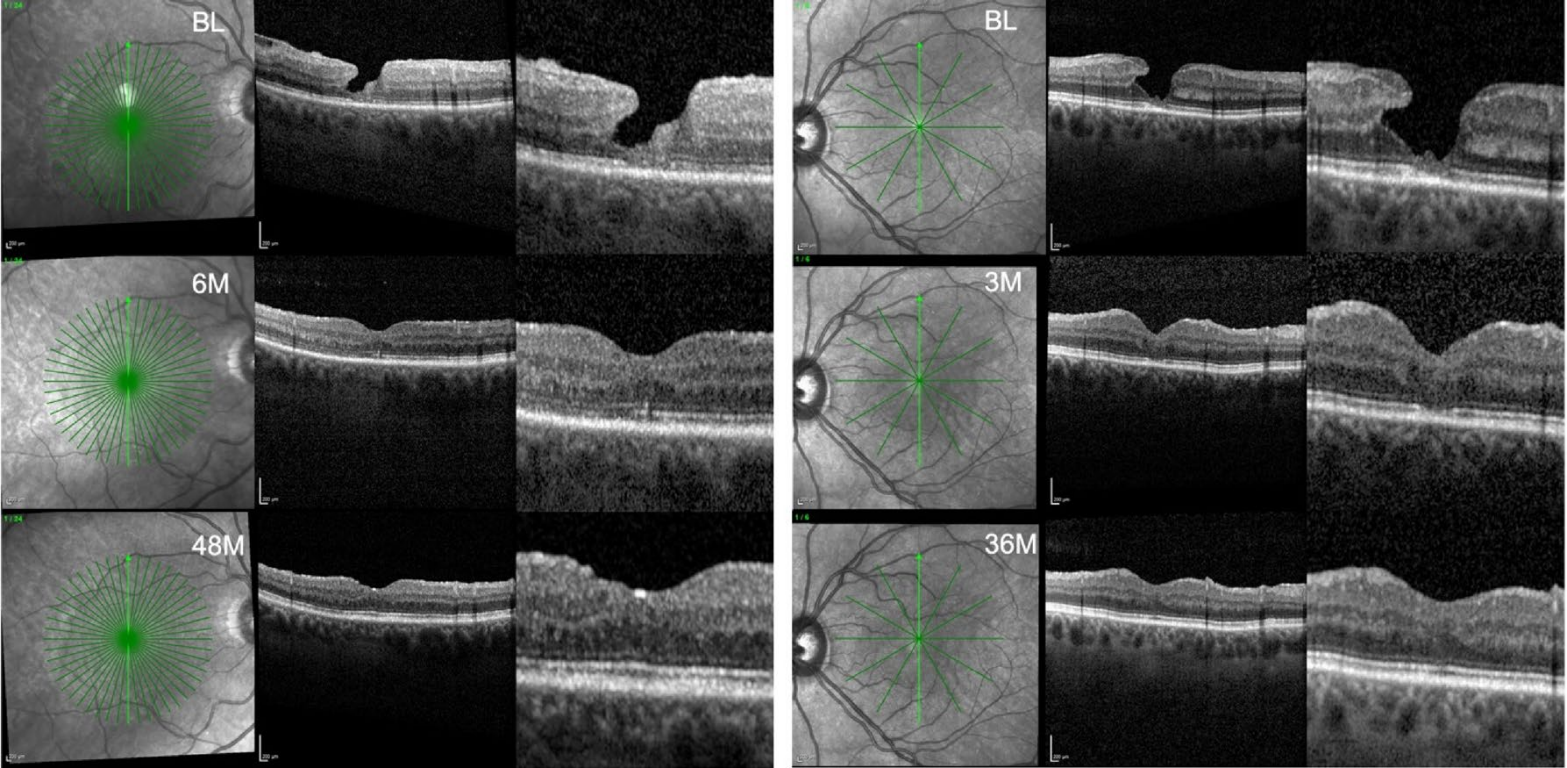
Exemplary depiction of seemingly complete regeneration of the ellipsoid zone in two patients with lamellar macular hole as demonstrated by Spectral Domain Optical Coherence Tomography (matched A- and B-Scan). Baseline (BL; top), within the first year (middle) and longest follow-up (bottom) Scan.

Initially, 16 out of 20 patients (80%) presented with an ellipsoid zone defect, with a mean initial defect width of 160 ± 74.77 µm (median: 159 µm**,** range: 58–316 µm). After a follow-up period of 16.8 ± 9.71 months (range: 6–36 months), complete EZ restoration was observed in 10 out of 16 cases (62.5%) (Fig. 3)**.** In 3 out of 16 cases, partial EZ regeneration was noted, while in 3 out of 16 cases, no improvement or even an increase in defect size was observed. The final mean defect width was 60.31 ± 104.67 µm (median: 0 µm**,** range: 0–359 µm), demonstrating a significant reduction in EZ defects over the entire follow-up period (*p* = 0.005**,** Wilcoxon signed-rank test).

19 out of 20 patients (95%) showed initially an epiretinal proliferation. After surgery all OCT scans showed an absence of ERP recurrence except for one case that relapsed after 24 months as mentioned before.

Preoperatively, the mean central retinal thickness (CRT) was 166.45 ± 42.4 µm (median: 156.00 µm**,** range: 112–230 µm). At the last follow-up visit, the CRT had increased to 232.8 ± 36.04 µm (median: 233.50 µm, range: 150–296 µm). This increase in CRT was statistically significant (*p *< 0.001, Wilcoxon signed-rank test).

Postoperatively, within 1–6 months, cystoid macular edema (CME) was observed in 5 out of 20 patients (25%). All patients initially received treatment with non-steroidal anti-inflammatory eye drops (0.3% Nepafenac). Only one patient experienced a relapse and was subsequently treated with a parabulbar injection of 40 mg triamcinolone. In two cases, the cystoid macular edema completely resolved, while in three cases, chronic intraretinal microcysts persisted. These microcysts were not considered visually significant and therefore did not require further treatment.

After surgery there were no cases showing a secondary full thickness macular hole (FTMH) nor an endophthalmitis.

### Functional outcome

The mean baseline best-corrected visual acuity (BCVA) for all patients (n = 20) was 0.36 ± 0.16 logMAR, with a median of 0.35 and a range of 0.2–0.7 logMAR. At the last follow-up, BCVA improved to 0.19 ± 0.24 logMAR, with a median of 0.15 and a range of −0.1 to 1.0 logMAR. This corresponds to a mean improvement of 0.17 logMAR, which was statistically significant (*p* = 0.005, Wilcoxon signed-rank test).

Compared to baseline, an improvement in BCVA of at least one line (mean: 2,5 lines) was observed in 15 patients (75%), while 1 patient (5%) showed no change. A decline in BCVA of one line was noted in 4 patients (20%). However, none of the patients experienced a loss of BCVA of 2 or more lines during the follow-up period.

In the subgroup of pseudophakic patients (n = 9), the mean baseline BCVA was 0.39 ± 0.11 logMAR, with a median of 0.4 and a range of 0.2–0.6 logMAR. At the final follow-up, BCVA was 0.29 ± 0.28 logMAR, with a median of 0.2 and a range of 0–1.0 logMAR. The mean improvement of 0.1 logMAR was not statistically significant (*p* = 0.204, Wilcoxon signed-rank test). Therefore, no significant improvement in BCVA was observed in the pseudophakic subgroup.

Spearman’s correlation showed a positive, not significant correlation (r = 0.404, *p* = 0.077) between baseline and longest BCVA, meaning a worse preoperative BCVA resulted in a worse postoperative BCVA. There was no significant correlation between baseline CRT and BCVA (r = 0.059, *p* = 0.805) nor between the last CRT and BCVA (r = −0.308, *p* = 0.186). A statistically highly significant negative correlation was observed between baseline ellipsoid zone defects and best-corrected visual acuity (BCVA) at the final follow-up (r =  −0.642, *p* = 0.007). This indicates that larger initial EZ defects were associated with worse BCVA outcomes at the last follow-up. The negative correlation suggests that the greater the extent of EZ disruption at baseline, the lower the visual function remained over time.

Microperimetric analysis (n = 16) revealed no statistically significant differences between preoperative and final follow-up measurements. Mean retinal sensitivity remained stable (23.25 ± 2.63 dB, median 23.80 dB, range 18.5–26.8 dB preoperatively vs. 23.08 ± 2.94 dB, median 24.50 dB, range 17.8–26.40 dB at final follow-up; *p* = 1.000, Wilcoxon signed-rank test). Fixation stability also remained unchanged, with P1 values of 73.44 ± 30.55% preoperatively and 67.73 ± 32.37% at final follow-up (*p* = 0.529), and P2 values of 89.75 ± 15.78% vs. 87.53 ± 17.89%, respectively (*p* = 0.678).

## Discussion

This prospective interventional case series provides evidence that pars plana vitrectomy with adjunct platelet rich plasma for progressive, symptomatic lamellar macular holes is both safe and effective in promoting anatomical and functional rehabilitation. Over a mean follow-up period of 40.2 ± 10.8 months, we observed significant improvements in central retinal thickness and ellipsoid zone integrity, as well as a statistically significant enhancement in best-corrected visual acuity and stable microperimetry results. As our cohort size is limited, the study therefore has a predominantly descriptive character and focuses on long-term outcomes of this surgical technique. Surgical intervention in combination with the use of PRP as an adjuvant might support long-term retinal remodeling and visual recovery in LMH patients.

BCVA improved significantly over the follow-up period, with 75% of patients (n = 20) showing a significant functional gain of 0.17 logMAR. A subgroup analysis revealed that in pseudophakic patients (n = 9) the mean improvement of 0.1 logMAR was not statistically significant. Nevertheless, these 24 months or longer results are consistent with previous 12-month findings, showing a VA gain of 0.15 logMAR^16^.

Similar postoperative visual acuity outcomes have also been reported in previous studies. For example, Parisi et al. in their meta-analysis of 13 studies on tractional and degenerative LMH described visual acuity improvement between 0.1 and 0.21 logMAR in degenerative LMH cases^9^. However, direct comparisons must be approached cautiously due to methodological differences. Some studies, such as Giansanti et al., who analysed 16 eyes with a 36-month follow-up period undergoing vitrectomy and ILM-peeling without using adjuvants, reported higher VA gains (0.3 logMAR) after phacovitrectomy, suggesting that lens status significantly influences outcomes^23^. To date the study of Chehaibou et al. is the largest cohort of LMH undergoing surgery (complete ILM/ERP-peeling or ILM/ERP-perihole sparing peeling) with a mean follow-up of 24 months with a VA gain of 0.11 logMAR^10^. Preoperative lens status is inhomogeneous distributed and reduces comparability to our study.

Our subgroup analysis among pseudophakic patients also reflects this influence, highlighting the need for larger cohorts to better understand the true functional impact of vitrectomy in LMH on the one hand and the additional effect of PRP on the other hand. Nevertheless, even in pseudophakic patients, functional improvements were observed, supporting the potential benefit of surgical intervention. The subgroup analysis of pseudophakic patients (n = 9) revealed a non-significant visual acuity gain of 0.1 logMAR compared to our 12-month data, where pseudophakic patients exhibited a significant improvement of 0.13 logMAR (*p* = 0.047)^17^. The lens status must be considered as a confounding factor in the analysis of BCVA. The significance of visual improvement differed between this study and the previous one due to variations in patient inclusion criteria (specifically the minimum follow-up period of 12 versus 24 months) and therefore resulting in dropouts, as well as the overall small cohort sizes (13 and 9 patients), respectively^17^. Given the limited cohort size, the statistical power of the findings remains restricted. The variance in visual acuity outcomes also differs in the literature, primarily due to short follow-up periods and small cohorts of pseudophakic patients^24^. In some studies, a pseudophakic subgroup is entirely absent^23^. Therefore, larger pseudophakic cohorts undergoing vitrectomy with extended follow-up periods are necessary to draw more robust conclusions and show the isolated effect of PRP in LMH surgery.

The exact influence of pre- /postoperative lens status respectively on functional outcomes remains indeterminate in this small cohort. Although the use of PRP is exploratory in our study and causality cannot be proven, our previous findings and evidence from other studies might suggest a potential functional advantage of surgical intervention for lamellar macular holes, even in pseudophakic patients^10,11,17^. The management of patients diagnosed with LMH will still be an ongoing matter of debate while some recommend surgical intervention only cautiously when visual and morphological deterioration occur to stabilise the LMH^25,26^, while others report about a functional benefit through surgical intervention only in ERM-Foveoschisis^24,26^. However, an increasing number of studies are emerging that demonstrate a functional and morphological benefit, further supporting the potential advantages of pars plana vitrectomy^6–8,15–17,27^. Consequently, the ongoing controversial debate regarding functional and morphological benefits of vitrectomy may gradually shift towards a more affirmative stance on surgical intervention for all progressive, symptomatic lamellar macular holes and might result in an evidence-based consensus in future.

However, in our study 5% of patients showed no change (n = 1), and another 20% exhibited a decline (n = 4) in visual acuity. In three of four patients the decline postoperatively was one line less compared to baseline visual acuity and couldn’t be correlated to the retinal anatomy. Thus, it was interpreted as an intraindividual fluctuation within the range of physiological variability. The fourth patient experienced a decline in visual acuity of 4 lines in the context of an advanced LMH preoperatively with a limited visual prognosis. This patient developed a CME—same clinical course as the vitrectomized fellow eye—indicating a potential predisposition to an exaggerated inflammatory response. The observed decrease in visual acuity is therefore more likely attributable to an individual-specific postoperative regenerative course with an ultimately unfavourable anatomical outcome.

Although BCVA and retinal anatomy improved significantly, microperimetric parameters were stable over the entire follow-up period. Importantly, the absence of deterioration in retinal sensitivity or fixation stability supports the safety of this surgical approach and shows that no iatrogenic retinal damage was induced. Furthermore, both measures capture different aspects of macular function, while BCVA reflects central high-contrast resolution, microperimetry provides additional topographic information on retinal sensitivity and fixation behaviour^28^. Moreover, microperimetry was only available in a subset of patients (n = 16, see Limitations), which may have limited the detection of subtle postoperative changes.

Beyond functional recovery, our results demonstrate significant morphological regeneration over the extended follow-up period. Notably, visual acuity outcomes are determined not only by the restoration of foveal architecture but, more importantly, by EZ integrity^29,30^.

Initially 80% of our participants presented with ellipsoid zone defects. Complete ellipsoid zone restoration was observed in 62.5% of those cases over 16.8 ± 9.71 months, with a significant reduction in mean defect width (baseline 160 ± 74.77 µm, last follow-up 60.31 ± 104.67 µm, *p* = 0,005). This finding is noteworthy, as EZ integrity is closely associated with visual function, as shown in previous studies^25,31,32^. Patients with larger baseline EZ defects had significantly worse BCVA at final follow-up, confirming EZ integrity as a key determinant of functional recovery. Nevertheless, restoration of the ellipsoid zone is a well-known phenomenon after conventional and PRP-assisted LMH-surgery but with limited data about the kinetics. While Hasebe et al. reported about EZ restoration up to nine months after FTMH surgery, we showed a continuous narrowing beyond the first postoperative year^33^. Using PRP as adjunct might further support foveal recovery after surgical intervention, but the exact contribution of PRP on EZ-regeneration and the temporal dynamics warrants further validation.

EZ defects may indicate advanced degeneration in LMH, suggesting that early surgical intervention could improve postoperative outcomes. This is supported by findings from Holland et al., who reported greater visual acuity improvement in patients with fewer preoperative EZ defects^34^. Nevertheless, our data demonstrated a high EZ regeneration rate despite advanced defects in 80% of participants, possibly attributable to PRP application. To proof causality further, larger studies with a randomised, controlled design are necessary.

Platelets are rich in growth factors and cytokines such as VEGF, EGF, PDGF and IGF-1 and IGF-2 among others^18^. The key player in foveal recovery are Mueller cells: LMH is characterized by partial loss of foveal tissue and disruption of Mueller cell cone^19^. These cavitations are replaced by epiretinal proliferation, a mid-reflective tissue composed of glial cells, fibroblasts and hyalocytes which act as glial scar tissue reconstructing the foveal defect^35^. Platelets become activated upon contact with disintegrated neuroretinal tissue following ILM-peeling, which removes the basal membrane of Mueller cell endfeet (‘surgical decapping’)^20^. This trigger signaling pathways in Mueller cells, promoting their proliferation, tissue remodeling, and migration. Further in-vivo studies are required to better understand the exact molecular regeneration mechanisms in LMH. Furthermore, Ricardi et al. recently suggested that also platelet concentration may influence LMH closure rates and foveal recovery^22^. Future studies are needed to determine an optimal platelet concentration. PRP might have a long-term regenerative potential in LMH, which is especially useful in advanced cases with EZ defects^14,17^.

Furthermore, our results show that vitrectomy stabilizes foveal morphology, with primary and long-lasting defect closure achieved in all cases. CRT increases immediately in the early postoperative days as the applied platelets cover the inner surface of the LMH in a patch-like manner. This effect is transient as the platelets are resorbed in the first weeks postoperatively. In the extended follow-up the increase in CRT is significant and possibly indicates structural recovery and halts the degenerative process. However, three patients (15%) developed recurrent foveal defects, including one without ILM peeling. This supports previous findings indicating that ILM peeling may play a crucial role in preventing LMH recurrence^15–17^. Pathophysiologically, ILM peeling removes residual posterior vitreous cortex, thereby eliminating tangential traction. This facilitates retinal surface relaxation, promoting foveal regeneration. Surgical decapping of the Mueller cells stimulates wound healing, which could be further supported by adjuvants like PRP. However, the quality of ILM peeling remains a critical factor, as poor technique may cause iatrogenic retinal damage and foveal thinning. Furthermore, interrupted ILM can contract, leading to gliotic processes, foveal fibrosis, and visual impairment^36^. Given this side effects and increased postoperative risks, the necessity of ILM-peeling has been questioned, leading to studies on fovea-sparing ILM peeling to preserve Mueller cell endfeet, crucial for cone physiology. The ILM may serve as a scaffold for Mueller cell migration into the defect, contributing to foveolar microstructure restoration. Ricardi et al. demonstrated “the no retina touch technique” in combination with platelet rich plasma to avoid iatrogenic trauma and reduce postoperative complications^22^. Given the small number of patients (n = 8) and short follow-up in that study, statistical power and evidence strength are limited, especially since extended follow-up is crucial to detect all changes. Morescalchi et al. demonstrated foveolar ILM non-peeling (1–2 disc diameters) with ERP removal compared to observation alone, reporting better visual acuity in the interventional group^11^. No postoperative complications such as FTMH, retinal detachment, or cystoid macular edema were reported during the 6-month follow-up. However, paracentral micro-scotomas were observed. The group of Ho et al. compared a group of conventional ILM-peeling with foveolar ILM-non-peeling leaving a 400 µm diameter ILM (foveolar size) with flat margins^12^. ERP was repositioned after trimming the edges and folding them into the macular hole. This surgical modification aims to seal the lamellar macular hole and should be critical in reconstructing the foveal depression as well as preserving the retinal layers. Compared to the conventional group, the foveolar ILM non-peeling group showed better BCVA, smaller retinal defects and a more restored EZ and a smoother foveal depression in an average follow-up time of 28 months.

Another determinant when modifying conventional pars plana vitrectomy for LMH is the presence and removal of ERP. In our study ERP was initially detected in almost all patients (95%), but only one case showed recurrence after surgery. Already intraoperatively the surgeon recognized a large amount of ERP which might have not been removed completely as the patient showed a re-opening of the LMH again with ERP. ERP can be seen as glial scar tissue that is formed as an attempt of the Mueller cells to heal the tissue defects and can cover the whole inner surface and connect to the conus to further stabilize the defect^19^. The impact of ERP presence on surgical outcome remains unclear. A meta-analysis of Xu et al. compared the surgical outcome of LMH with and without ERP and found a greater postoperative increase in VA in eyes without ERP^37^. Some studies reported worse postoperative VA and more EZ disruption after conventional ILM peeling in LMH with ERP^26,38^. Lai et al. and others reported similar VA improvements independent of ERP presence^7,25,39,40^. Since ERP was present in 95% of our participants, we could not statistically evaluate these findings. Nevertheless, only one ERP recurrence occurred postoperatively after 24 months, suggesting that ERP peeling halts degeneration and promotes foveal healing.

In general, modifications to conventional pars plana vitrectomy and the use of adjuvants appear beneficial, but further studies with longer follow-up and larger cohorts are needed to draw more robust, causative conclusions. The surgical approach should be individualized based on preoperative OCT biomarkers such as ILM peeling necessity, presence of ERP, EZ integrity, CRT, and baseline VA. Developing a standardized scoring system to assess LMH progression could help guide the selection of the most appropriate surgical technique in the future. Nonetheless, our findings suggest that ILM peeling is crucial as recurrences only occurred in patients without ILM peeling. ILM- and ERP-peeling can remove tractional forces and halt degeneration, potentially leading to stable foveal regeneration and improved visual acuity. Since the 1990s, PRP has been established as an adjuvant therapy in vitrectomy for full-thickness macular holes, consistently demonstrating efficacy and safety^41,42^. Considering our cohort (progressive LMH, 80% with EZ defects, baseline BCVA 0.36 ± 0.16 logMAR) and postoperative outcomes, PRP might be beneficial also in these types of macular lesions.

Transient complications, such as the postoperative incidence of cystoid macular edema in 25% of patients, were successfully treated in most cases using non-steroidal anti-inflammatory eye drops. Only one patient required additional treatment with parabulbar triamcinolone. Two cases resolved completely, whereas chronic intraretinal microcysts persisted in three patients at final follow-up without affecting visual acuity negatively. Interpretation of these findings is challenging as the cohort size is small, three of those five patients underwent combined phacovtrectomy and those findings could also be attributed to advanced LMH stages with chronic degeneration. However, a PRP-induced inflammation cannot be entirely excluded as Gamulescu et al. described one casae with an inflammatory reaction to PRP leading to exudative retinal detachment after vitrectomy^43^. To our knowledge, this remains the only report of platelet-related side effects in intraocular surgery.

Importantly, no cases of severe visual-threatening complications such as full-thickness macular hole formation, vitreous hemorrhage, retinal tears, rhegmatogenous retinal detachment or endophthalmitis were observed**,** confirming the favorable safety profile of vitrectomy for LMH with PRP.

Reports in the literature indicate that the incidence of secondary full-thickness macular hole formation after vitrectomy for LMH can be as high as 27.7%^9^. Higher incidence is particularly noted in LMH cases associated with epiretinal proliferation. A recent study by Chehaibou et al. reported that 9% of vitrectomized eyes developed full-thickness macular holes. Pseudophakia and ILM-peeling were identified as protective factors, whereas EZ defects were considered risk factors^10^. In our study, no cases of secondary full-thickness macular hole formation or other iatrogenic complications occurred, even after extended follow-up (median 42 months). This despite including patients with advanced LMH stages, indicated by 80% EZ defects and potentially reduced foveal stability. Additionally, over 50% of the patients underwent phacovitrectomy. This may be partly attributable to the surgical expertise. Additionally, PRP may enhance LMH surgery safety by stabilizing retinal structures and promoting tissue integrity.

It remains elusive whether modifying the surgical approach really reduces the risk of postoperative complication or whether the modification is well tailored to the individual preoperative biomarkers.

While anatomical and functional improvements observed in this series are noteworthy due to the extended follow-up period, the small cohort size and lack of a control group do not permit causal conclusions regarding the contribution of PRP. In the absence of a non-PRP comparison group, the potential regenerative role of PRP cannot be isolated from the effects of vitrectomy and ILM-peeling alone. Our interpretation is exploratory rather than confirmatory. Larger, randomized controlled trials with long-term follow-up are needed to compare different surgical techniques and to further proof our associations. Especially larger pseudophakic cohorts are needed to proof the benefit of LMH surgery. Follow-up adherence declined over time due to the long follow-up period, increasing age or other personal reasons. As a result, longitudinal comparisons should be interpreted with caution due to the potential for follow-up bias and inhomogeneous sample size at different time points. Though, any patient postoperatively had a mean number of 4 visits out of possible 7 during the 60-month follow-up period. Additionally, microperimetry data were available for only 16 out of 20 patients. This limitation is due to the clinical practice, where access to this specialized diagnostic procedures may be restricted by personnel- and time-dependent availability. Furthermore, lens status must be considered as possible confounder. Due to the small sample size of pseudophakic patients this subgroup analysis is of limited statistical power. Furthermore, as LMH is a rare disease larger, controlled randomized multicenter studies will be required to directly compare surgical techniques and to determine whether PRP confers a measurable therapeutic benefit in LMH surgery.

## Conclusion

In conclusion, we demonstrated that pars plana vitrectomy with adjunct PRP is safe and could provide favorable long-term anatomical and functional outcomes, even in advanced LMH. Extended follow-up over several years is essential to capture possible regeneration mechanisms, particularly ultrastructural EZ restoration, which progresses slowly but steadily. PRP may improve the procedure’s safety by reducing the risk of secondary full-thickness macular hole formation. In advanced symptomatic LMH with EZ defects, PRP may enhance regenerative potential, but this needs further evidence. Randomized controlled multi-center trials with larger populations are needed to evaluate adjuvant therapies and refine surgical protocols for LMH treatment.

## Methods

### Study design

In this prospective interventional case series 20 eyes of 20 patients were included with symptomatic, progressive LMH based on the diagnostic criteria according to Hubschmann et. al.^1^. All eyes underwent 23-gauge pars plana vitrectomy with the application of adjunct autologous, highly concentrated platelet- rich plasma. Surgery was conducted by highly experienced vitreoretinal surgeons (S.G.P., T.C.K.) at the Department of Ophthalmology, LMU University Hospital, Munich between December 2019 and March 2025. This study was approved by the institutional review board of the University Eye Hospital of the Ludwig-Maximilians-University and was performed in agreement with the ethical principles of the Declaration of Helsinki. All patients gave written informed consent prior to undergoing the interventions outlined in the manuscript.

### Patient selection

The criteria for a SD-OCT based diagnosis of LMH were defined according to Hubschmann et. al.: 1) irregular foveal contour, 2) foveal cavity with undermined edges and, 3) signs of tissue loss^1^. Patients were recommended to undergo surgery when at least 2 of the findings occurred during the preoperative follow-up period 1) significant reduction in visual acuity, 2) progression of the morphological findings 3) significant impairment of quality of life caused by metamorphopsia. Those cases were considered progressive LMH. Included were all patients that met the consensus-based diagnosis criteria as mentioned above, fulfilled at least 2 of the 3 recommendations for surgery and had a follow-up of at least 24 months postoperatively. Until March 2025 47 patients with lamellar macular hole were operated according to the study protocol mentioned above. Only 20 eyes of 20 patients reached a minimum follow-up period of 24 months and therefore underwent further analysis in this study. Standard scheduled procedure in our clinic is an assessment after 3, 6, 12, 24, 36, 48 and 60 months postoperatively.

The exclusion criteria were concomitant retinal pathologies such as diabetic retinopathy, vitreous hemorrhage, retinal detachment, age-related macular degeneration, inflammatory disease, vascular occlusion, high myopia ≥ -6 dpt or trauma.

Each patient was evaluated pre- and postoperatively after 3, 6, 12, 24, 36, 48 and 60 months. During each visit a complete eye examination was performed and consisted of slit lamp biomicroscopy with dilated fundus examination, SD-OCT scans with volume and radial scans (SPECTRALIS HRA + OCT, Heidelberg Engineering, Heidelberg, Germany) and microperimetry (MAIA, Centervue Inc., Fremont, CA, USA). Best-corrected visual acuity was determined by using standard ETDRS charts at 4 m after subjective manifest refraction had been measured. Postoperative complications were recorded at any time point during the follow-up period.

### PRP preparation

The preparation of PRP followed the methodology outlined in previous studies^15–17^. A total of 105 mL of whole blood was collected and anticoagulated at a ratio of 1:7. The blood components were then separated into platelet-rich plasma, red blood cells, and platelet-poor plasma using a specialized closed-circuit centrifugation system (Arthrex Angel System™, Arthrex, Naples, FL, USA). This highly concentrated PRP shows a reduced proportion of pro-inflammatory leukocytes and an 8.8-fold higher platelet concentration compared to whole blood.

### Surgical procedure

The vitrectomy was performed using a 23-/25-gauge pars plana approach by experienced surgeons. Posterior vitreous detachment was induced, followed by peeling of epiretinal tissue if present and the internal limiting membrane, except one case. Whenever epiretinal tissue was present, it was removed completely. No surgical modifications like trimming, repositioning or embedding were applied. The ILM was peeled in all cases up to the major vascular arcades. MembraneBlue-Dual dye (0.125 mg Brilliant Blue G and 0.75 mg Trypan Blue, D.O.R.C., Zuidland, Netherlands) was used for staining, and internal limiting membrane peeling was performed with at least one additional control staining.

Combined phacovitrectomy with implantation of a pre-calculated intraocular lens was performed in all phakic patients. After completing the vitrectomy, highly concentrated platelet-rich plasma (0.1 mL) was applied to the posterior pole under air or gas tamponade (SF6, C2F6). Table S1 gives a detailed per-eye procedural overview. Patients were strongly advised to maintain a supine position for at least two hours postoperatively to optimize platelet-rich plasma distribution.

### Main outcome measures

Primary outcome measure was defect closure and postoperative morphology on SD-OCT, such as integrity of the inner and outer retinal layers and the inner foveal depression on all follow-up scans over a minimum follow-up of 24 months and up to 60 months. As secondary outcome postoperative BCVA and CRT, modifications in EZ-defect width, microperimetry (retinal sensitivity, fixation stability) and prevalence of complications were included. A disruption of the EZ was defined by a loss of continuity of these retinal layers on the SD-OCT B-scan measured with the calliper tool parallel to the retinal pigment epithelium. Any change in EZ defect length over the follow-up period was defined as improvement or impairment. CRT was defined as smallest vertical diameter on SD-OCT B-Scan and measured with the calliper tool over the follow-up time.

### Statistical analysis

The statistical analysis was carried out by IBM SPSS Version 29 (IBM Corporation, New York, NY, USA). Numerical data are presented as mean ± standard deviation, median and range, whereas qualitative variables were displayed as frequencies (absolutes) and percentages (%). The correlation between two continuous, non-normally distributed data were calculated with the Spearman correlation. Normal distribution was tested with the Shapiro Wilk Test. To compare two related groups Wilcoxon signed-rank test was used. A p-value of less than 0.05 was considered statistically significant.

## Supplementary Information

Below is the link to the electronic supplementary material.

Supplementary Material 1

## Data Availability

The datasets and analysis are available from the corresponding author on reasonable request due to reasons of sensitivity.
