# Supplementary material for: Extended follow-up of ultrastructural remodeling and functional recovery after lamellar macular hole surgery using autologous platelet-rich plasma
Source: Sci Rep. 2026 Jun 24;16:19565. doi: 10.1038/s41598-026-58252-0 (PMC13294354; doi:10.1038/s41598-026-58252-0)
Supplement: Supplementary file 1 — Supplementary Material 1 [file 41598_2026_58252_MOESM1_ESM.docx]

Supplements

| **ID** | **Tamponade** | **Surgeon** | **Recurrence** | **Recurrence** [months] |
| --- | --- | --- | --- | --- |
|  |  |  | [y=1, n=0] |  |
| 1 | Air | P | 0 | - |
| 2 | SF6 | K | 1 | 12 |
| 3 | C2F6 | P | 0 | - |
| 4 | SF6 | K | 0 | - |
| 5 | SF6 | K | 0 | - |
| 6 | SF6 | K | 0 | - |
| 7 | Air | P | 0 | - |
| 8 | Air | P | 1 | 24 |
| 9 | Air | K | 0 | - |
| 10 | Air | P | 1 | 0 |
| 11 | Air | P | 0 | - |
| 12 | Air | P | 0 | - |
| 13 | SF6 | K | 0 | - |
| 14 | Air | K | 0 | - |
| 15 | C2F6 | P | 0 | - |
| 16 | Air | P | 0 | - |
| 17 | Air | P | 0 | - |
| 18 | Air | P | 0 | - |
| 19 | Air | P | 0 | - |
| 20 | Air | P | 0 | - |

Table S1: Detailed per-eye surgical data
